# A nomogram integrating machine learning with clinical predictors for osteosarcopenia risk prediction in type 2 diabetes mellitus

**DOI:** 10.3389/fendo.2026.1876521

**Published:** 2026-07-15

**Authors:** Dan Liang, Zhenrun Zhan, Yongze Zhang, Sunjie Yan

**Affiliations:** 1 Department of Endocrinology, The First Affiliated Hospital, Fujian Medical University, Fuzhou, China; 2 Department of Endocrinology, National Regional Medical Center, Binhai Campus of the First Affiliated Hospital, Fujian Medical University, Fuzhou, China; 3 Clinical Research Center for Metabolic Diseases of Fujian Province, The First Affiliated Hospital, Fujian Medical University, Fuzhou, China; 4 Fujian Key Laboratory of Glycolipid and Bone Mineral Metabolism, The First Affiliated Hospital, Fujian Medical University, Fuzhou, China; 5 Diabetes Research Institute of Fujian Province, The First Affiliated Hospital, Fujian Medical University, Fuzhou, China; 6 Metabolic Diseases Research Institute, The First Affiliated Hospital, Fujian Medical University, Fuzhou, China

**Keywords:** nomogram, obesity, osteosarcopenia, sarcopenia, type 2 diabetes mellitus

## Abstract

**Objective:**

Type 2 diabetes mellitus (T2DM) predisposes patients to osteosarcopenia, a debilitating condition characterized by concurrent bone loss and muscle wasting. This study aimed to develop and internally validate a nomogram for predicting osteosarcopenia risk in T2DM patients aged ≥ 40 years.

**Methods:**

The test cohort included 5,412 hospitalized T2DM patients (January 2010–July 2024), and the temporal validation cohort included 1,671 patients (August 2024–December 2025) from the First Affiliated Hospital of Fujian Medical University. Logistic regression and machine learning algorithms (Boruta, random forest, LASSO) were combined for feature selection. The nomogram was constructed via multivariable logistic regression. We carried out receiver operating characteristic (ROC) curve analysis, calibration, decision curve analysis (DCA), and bootstrap validation for assessing the nomogram. Restricted cubic splines were employed for exploring potential nonlinear associations.

**Results:**

Eight independent predictors, which encompassed gender, age, BMI, WHtR, fracture history, diabetic foot ulcer (DFU), smoking status, and diabetic kidney disease (DKD), were identified. These predictors were incorporated into the nomogram. The nomogram achieved AUCs of 0.864 and 0.904 in the test cohort and validation cohort, respectively. Accordingly, favorable calibration and positive net benefit on DCA was demonstrated. Higher BMI served as a protective factor (OR = 0.56, 95% CI: 0.53–0.59). Besides, higher WHtR acted as a risk factor (OR = 1.47, 95% CI: 1.28–1.69). Restricted cubic spline analysis revealed a significant negative nonlinear relationship between BMI and osteosarcopenia risk, and a significant positive nonlinear relationship between WHtR and osteosarcopenia risk.

**Conclusion:**

This nomogram, based on eight readily available clinical variables, exhibits excellent discriminative performance and clinical utility for predicting osteosarcopenia risk in T2DM patients aged ≥ 40 years. Further multicenter external validation is warranted.

## 1 Introduction

Type 2 diabetes mellitus (T2DM) constitutes a growing global health burden, with its prevalence projected to increase substantially in the upcoming decades (1). In addition to its well-documented microvascular and macrovascular complications, T2DM exerts profound adverse effects on the musculoskeletal system, elevating the risk of osteoporosis, sarcopenia, and their co-occurrence, a clinical condition defined as osteosarcopenia (2, 3). Such musculoskeletal deterioration clinically presents as increased vulnerability to fragility fractures, falls, and functional deterioration, which substantially impairs the quality of life and survival of affected patients (4).

Epidemiological evidence from a meta-analysis indicates that up to 28% of individuals with T2DM satisfy the diagnostic criteria for sarcopenia (5). Among community-dwelling populations, the prevalence of osteosarcopenia increases significantly with age, rising from 14.3% to 59.4% in males and from 20.3% to 48.3% in females between the age groups of 60–64 years and ≥75 years (6). The pathogenesis of osteosarcopenia in T2DM is multifactorial and complex, with core underlying mechanisms including insulin resistance, chronic inflammation, advanced glycation end product (AGE) accumulation, and disrupted muscle-bone crosstalk (7). Insulin resistance triggers skeletal muscle atrophy by inhibiting PI3K/Akt-mediated protein synthesis and activating FoxO-driven protein degradation (8). Chronic low-grade inflammation in T2DM, characterized by elevated levels of TNF-α and IL-6, induces muscle wasting via NF-κB activation and promotes bone loss by facilitating osteoclastogenesis and suppressing osteoblastic function (9, 10). Furthermore, excessive accumulation of AGE in bone collagen and muscle tissue directly impairs bone material properties and myotube differentiation, and disrupts muscle-bone signal transduction, thereby accelerating the simultaneous degeneration of skeletal bone and muscle tissues (11, 12).

Body mass index (BMI) and waist-to-height ratio (WHtR) are two accessible anthropometric indices for patients with T2DM, yet their modulatory roles in musculoskeletal health exhibit prominent complexity and a distinct paradox. As a conventional indicator of general adiposity, BMI is traditionally recognized to be positively correlated with higher bone mineral density and reduced fracture risk (13, 14). However, this conventional perspective is challenged by the well-documented “obesity paradox” in T2DM populations, in which patients with relatively high BMI still suffer from severe muscle wasting and increased bone fragility (15, 16). In contrast, WHtR, a sensitive biomarker of central obesity, outperforms BMI in reflecting visceral fat accumulation. Elevated WHtR induces systemic inflammation, insulin resistance, and lipotoxicity, which in turn suppress muscle protein synthesis and disturb bone remodeling processes (17, 18). Although emerging evidence demonstrates that WHtR is a more powerful predictor of sarcopenia and osteoporosis risk than BMI in T2DM patients (19), few studies have incorporated both indices into an integrated predictive model, and the nonlinear relationships of these two anthropometric measures with musculoskeletal outcomes remain incompletely clarified.

Current risk prediction tools for osteosarcopenia in the T2DM population are limited. The FRAX tool has been shown to significantly underestimate fracture risk in patients with diabetes (20), and frequently fails to capture the excess disease-related risk in this patient population. Therefore, there is a pressing need to develop a practical and accurate prediction model specifically tailored for the T2DM population to enable the early identification of individuals at risk of osteosarcopenia. Such a tool, constructed based on routinely collected clinical variables, could facilitate targeted screening and timely clinical intervention, thereby ultimately reducing the disease burden attributable to fractures and subsequent disability. The present study aimed to develop and internally validate a nomogram-based prediction model for osteosarcopenia in Chinese patients with T2DM aged 40 years and older.

## 2 Materials and methods

### 2.1 Patient data

Patients hospitalized at the First Affiliated Hospital of Fujian Medical University from January 2010 to July 2024 were enrolled as the test cohort. Additionally, a temporal validation cohort was recruited from August 2024 to December 2025 at the same institution. This study was approved by the Ethics Committee of the First Affiliated Hospital of Fujian Medical University (Approval reference: MRCTA, ECFAH of FMU [2017]131). Written informed consent was obtained from all participants. Participants were eligible for inclusion if they met all the following criteria: (1) age ≥ 40 years; (2) a confirmed diagnosis of T2DM in accordance with the diagnostic criteria established by the American Diabetes Association (21); (3) complete body composition and bone mineral density (BMD) data measured via dual-energy X-ray absorptiometry (DXA) were available. Participants were excluded from the analysis if they met any of the following criteria: (1) a diagnosis of type 1 diabetes mellitus, gestational diabetes mellitus, or other specific types of diabetes; (2) a medical history of severe systemic diseases, including advanced malignancy, severe hepatic dysfunction, thyroid dysfunction, or stroke; (3) a history of major trauma or surgery within the preceding six months that could substantially affect musculoskeletal health; (4) baseline missing data exceeding 20% for any key predictor variable included in this study. Ultimately, a total of 5,412 eligible patients with T2DM were included in the final analysis of the test cohort, and 1,671 patients were enrolled in the validation cohort.

### 2.2 Definition of osteosarcopenia

Osteosarcopenia is defined as the concurrent presence of osteopenia/osteoporosis and sarcopenia (2). Osteopenia and osteoporosis are combined into a single osteosarcopenia outcome since both represent a continuum of systemic skeletal disorders with impaired bone microarchitecture and reduced BMD. These disorders, result in increased bone fragility and fracture susceptibility (2), particularly in T2DM patients (22). osteopenia/osteoporosis is diagnosed through DXA based on the WHO criteria. The criteria are elucidated as osteopenia (T-score between −2.5 and −1.0 at any site of the lumbar spine, left femoral neck, left hip, or whole body) and osteoporosis (T-score ≤ −2.5) (23). The appendicular muscle mass index (ASMI) serves as a measure of appendicular skeletal muscle mass (ASM) divided by height squared. For men and women, the ASMI cutoff points for sarcopenia diagnosis are 7.0 kg/m^2^ and 5.4 kg/m^2^, respectively (24). Low muscle strength is defined as a dominant grip strength of < 28 kg in men and < 18 kg in women (24). In accordance with the 2025 consensus of the Asian Working Group for Sarcopenia (AWGS) (25), sarcopenia is diagnosed when both low muscle mass and low muscle strength are present.

### 2.3 Data collection

The following demographic and clinical variables were collected from each participant: age, gender, dominant grip strength (kg), body mass index (BMI, kg/m²), height (cm), waist circumference (cm), weight (kg), systolic blood pressure (SBP, mmHg), diastolic blood pressure (DBP, mmHg), heart rate (HR, bpm), fasting plasma glucose (FBG, mmol/L), glycated hemoglobin (HbA1c, %), lipid profiles including triglycerides (TG, mmol/L), low-density lipoprotein cholesterol (LDL-C, mmol/L), and high-density lipoprotein cholesterol (HDL-C, mmol/L), renal function markers (blood urea nitrogen [BUN, mmol/L], creatinine [Cr, mmol/L], and urinary albumin-to-creatinine ratio [UACR, mg/g], estimated glomerular filtration rate [eGFR, mL/min/1.73 m²]), liver function indicators (alanine aminotransferase [ALT, U/L] and albumin [Alb, g/L]), mineral metabolism parameters (serum calcium [Ca, mmol/L] and alkaline phosphatase [ALP, U/L]), and uric acid (UA, mmol/L). Collected comorbidities included hypertension, coronary heart disease (CHD), prior fracture, diabetic retinopathy (DR), diabetic peripheral neuropathy (DPN), and diabetic foot ulcer (DFU). Additionally, current medication use (metformin, sulfonylureas [SUs], non-sulfonylurea drugs [NSUs], α-glucosidase inhibitors, thiazolidinediones, insulin, dipeptidyl peptidase-4 inhibitors [DPP-4i], sodium-glucose cotransporter 2 inhibitors [SGLT2i], glucagon-like peptide-1 receptor agonists [GLP-1RA], and statins) and lifestyle factors (smoking and alcohol drinking status) were recorded. Diabetic kidney disease (DKD) is defined as a persistent reduction in eGFR (< 60 mL/min/1.73 m²) and/or persistent albuminuria (UACR ≥ 30 mg/g) lasting for more than 3 months, after excluding other causes of kidney injury (26, 27).

### 2.4 Statistical analysis

For variables with missingness below 20%, multiple imputation by chained equations was performed using the mice package (version 3.14.0). Baseline characteristics were compared between patients with and without osteosarcopenia. Continuous variables were compared via the Wilcoxon rank-sum test, and categorical variables via the chi-square test or Fisher’s exact test, as appropriate. Normally distributed data are presented as mean ± standard deviation (SD), and non-normally distributed data as median (IQR). Multicollinearity across covariates was assessed using the variance inflation factor (VIF); a VIF threshold of < 5 indicated no significant collinearity. We carried out multivariable logistic regression for identifying factors showing an independent association with osteosarcopenia. The results are expressed as odds ratios (ORs) with 95% confidence intervals (CIs). Model 1 incorporated BMI and Z-score standardized WHtR (z-WHtR) as the primary exposure variables. Model 2 incorporated BMI, z-WHtR, gender, age, SBP, DBP, and HR. Model 3 was the fully adjusted model incorporating BMI, z-WHtR, gender, age, SBP, DBP, HR, TG, LDL-C, HDL-C, ALT, UA, Alb, ALP, Ca, FBG, HbA1c, hypertension, CHD, fracture, DKD, DR, DPN, DFU, metformin, sulfonylureas, non-sulfonylurea agents, glucosidase inhibitors, thiazolidinediones, DPP-4i, SGLT2i, GLP-1RA, insulin, statins, smoking status, and alcohol consumption. Logistic regression with restricted cubic splines (RCS) was applied to examine potential nonlinear relationships between WHtR, BMI, and osteosarcopenia, with adjustment for all covariates. A random forest classifier with 1,000 trees was trained; feature importance was quantified using the mean decrease in the Gini index and stabilized through 10-fold cross-validation. The Boruta algorithm (1,000 trees) was further applied to screen relevant predictors by comparing each variable’s importance against that of random shadow variables. Model discrimination performance was evaluated using the area under the receiver operating characteristic (ROC) curve (AUC). Model calibration was assessed via calibration curves. Decision curve analysis (DCA) was performed to quantify clinical net benefit. Model performance was further evaluated in a temporal validation cohort, with AUC, calibration curves, and DCA likewise computed. A two-sided p-value < 0.05 was defined as statistically significant. All statistical analyses were carried out in R software (version 4.5.0).

## 3 Results

### 3.1 Patient characteristics

A total of 5,412 patients with T2DM aged ≥40 years were enrolled in the test cohort. Among these patients, 890 (16.4%) presented with osteosarcopenia (**Table 1**). Compared with patients without osteosarcopenia, those with osteosarcopenia were more likely to be male (63.9% vs. 31.6%, p < 0.001), older (65 ± 11 vs. 61 ± 10 years, p < 0.001), and exhibited lower levels of BMI (21.3 vs. 24.9 kg/m², p < 0.001), WHtR (0.51 vs. 0.55, p < 0.001), Alb (38.6 vs. 40.0 g/L, p < 0.001), and Ca (2.21 vs. 2.24 mmol/L, p < 0.001), but a higher HR (81 vs. 80 bpm, p = 0.002). In terms of comorbidities and complications, the osteosarcopenia group had higher proportions of fracture (9.3% vs. 7.2%, p = 0.025), DFU (6.4% vs. 3.4%, p < 0.001), and SGLT2i use (52.0% vs. 47.9%, p = 0.024), as well as lower proportions of hypertension (49.4% vs. 54.0%, p = 0.012) and metformin use (55.1% vs. 62.0%, p < 0.001) were achieved. Notably, smoking (35.4% vs. 24.8%, p < 0.001) and alcohol drinking (14.5% vs. 11.1%, p = 0.004) turned out to be more prevalent. We did not observe any significant differences in LDL-C, HDL-C, BUN, FBG, HbA1c, Cr, diabetes duration, DKD, diabetic retinopathy, DPN, or other antidiabetic medications (all p > 0.05). **Supplementary Table 1** lists the baseline characteristics of the validation cohort (n = 1,671).

**Table 1:** Baseline characteristics of T2DM patients in the test cohort.

Characteristic	Osteosarcopenia			P-value^2^
	Overall N = 5,412^1^	No N = 4,522^1^ (83.6%)	Yes N = 890^1^ (16.4%)	
Gender				<0.001
Female	2,552 (47.2%)	2,231 (49.3%)	321 (36.1%)	
Male	2,860 (52.8%)	2,291 (50.7%)	569 (63.9%)	
Age (year)	62 ± 10	61 ± 10	65 ± 11	<0.001
ASMI (kg/m^2^)	6.73 (6.03, 7.51)	6.96 (6.18, 7.68)	5.93 (5.19, 6.57)	<0.001
Grip strength (kg)	28 (20, 34)	28 (21, 35)	17 (14, 21)	<0.001
Waist circumference (cm)	87 (81, 93)	88 (82, 94)	82 (76, 88)	<0.001
Height (cm)	160 ± 8	160 ± 8	160 ± 9	0.080
BMI (kg/m^2^)	24.3 (22.1, 26.6)	24.9 (22.9, 27.2)	21.3 (19.7, 23.0)	<0.001
WHtR	0.54 (0.51, 0.58)	0.55 (0.51, 0.59)	0.51 (0.48, 0.55)	<0.001
SBP (mmHg)	137 ± 20	137 ± 20	135 ± 20	0.021
DBP (mmHg)	79 ± 11	79 ± 11	77 ± 10	<0.001
HR (bpm)	80 ± 12	80 ± 12	81 ± 12	0.002
UACR (mg/g)	13 (6, 44)	13 (6, 44)	14 (6, 47)	0.352
Triglycerides (mmol/L)	1.42 (0.98, 2.15)	1.46 (1.01, 2.21)	1.22 (0.86, 1.81)	<0.001
Total cholesterol (mmol/L)	4.50 (3.75, 5.33)	4.53 (3.77, 5.35)	4.37 (3.68, 5.21)	0.004
ALT (U/L)	20 (15, 30)	21 (15, 30)	19 (13, 28)	<0.001
LDL-C (mmol/L)	2.76 (2.13, 3.49)	2.78 (2.15, 3.49)	2.71 (2.07, 3.47)	0.179
HDL-C (mmol/L)	1.08 (0.90, 1.32)	1.08 (0.90, 1.31)	1.12 (0.91, 1.38)	0.001
Blood urea nitrogen (U/L)	5.45 (4.40, 6.81)	5.46 (4.42, 6.80)	5.38 (4.21, 6.86)	0.091
Uric acid (μmol/L)	310 (251, 375)	314 (255, 379)	287 (233, 354)	<0.001
Albumin(g/L)	39.8 (36.9, 42.5)	40.0 (37.2, 42.6)	38.6 (35.2, 41.4)	<0.001
Alkaline phosphatase (U/L)	72 (60, 89)	72 (59, 88)	76 (62, 95)	<0.001
Ca (mmol/L)	2.23 (2.15, 2.32)	2.24 (2.15, 2.32)	2.21 (2.13, 2.30)	<0.001
Fasting blood glucose (mmol/L)	7.7 (5.7, 10.8)	7.7 (5.8, 10.8)	7.6 (5.5, 10.9)	0.147
HbA1c (%)	8.80 (7.20, 10.70)	8.80 (7.10, 10.70)	9.00 (7.30, 11.00)	0.064
Creatinine (μmol/L)	61 (50, 75)	61 (50, 75)	60 (49, 76)	0.443
eGFR (mL/min/1.73m²)	101 (90, 109)	101 (91, 109)	100 (89, 109)	0.065
Hypertension, n (%)	2,884 (53.3%)	2,444 (54.0%)	440 (49.4%)	0.012
Coronary heart disease, n (%)	179 (3.3%)	150 (3.3%)	29 (3.3%)	0.929
Fracture, n (%)	407 (7.5%)	324 (7.2%)	83 (9.3%)	0.025
Duration of DM (years)	8.0 (3.0, 13.0)	8.0 (3.0, 13.0)	8.0 (3.0, 13.0)	0.957
Diabetic retinopathy, n (%)	1,304 (24.1%)	1,099 (24.3%)	205 (23.0%)	0.418
DPN, n (%)	2,441 (45.1%)	2,020 (44.7%)	421 (47.3%)	0.149
DFU, n (%)	211 (3.9%)	154 (3.4%)	57 (6.4%)	<0.001
Diabetic kidney disease, n (%)	1,768 (32.7%)	1,459 (32.3%)	309 (34.7%)	0.154
Metformin, n (%)	3,294 (60.9%)	2,804 (62.0%)	490 (55.1%)	<0.001
Sulfonylureas, n (%)	2,472 (45.7%)	2,073 (45.8%)	399 (44.8%)	0.580
Non-sulfonylurea drugs, n (%)	969 (17.9%)	824 (18.2%)	145 (16.3%)	0.170
Glucosidase Inhibitors, n (%)	2,297 (42.4%)	1,909 (42.2%)	388 (43.6%)	0.447
Thiazolidinediones, n (%)	575 (10.6%)	488 (10.8%)	87 (9.8%)	0.368
DPP-4i, n (%)	866 (16.0%)	733 (16.2%)	133 (14.9%)	0.346
SGLT2i, n (%)	2,629 (48.6%)	2,166 (47.9%)	463 (52.0%)	0.024
GLP-1RA, n (%)	999 (18.5%)	848 (18.8%)	151 (17.0%)	0.209
Insulin, n (%)	1,782 (32.9%)	1,497 (33.1%)	285 (32.0%)	0.530
Statins, n (%)	1,191 (22.0%)	999 (22.1%)	192 (21.6%)	0.733
Smoking, n (%)	1,438 (26.6%)	1,123 (24.8%)	315 (35.4%)	<0.001
Drinking, n (%)	630 (11.6%)	501 (11.1%)	129 (14.5%)	0.004
BMD category, n (%)				<0.001
Normal	2,393 (44.2%)	2,393 (52.9%)	0 (0.0%)	
Osteopenia	1,990 (36.8%)	1,439 (31.8%)	551 (61.9%)	
Osteoporosis	1,029 (19.0%)	690 (15.3%)	339 (38.1%)	
Sarcopenia, n (%)	1,385 (25.6%)	495 (10.9%)	890 (100.0%)	<0.001

^1^ n (%); Mean ± SD; Median (Q1, Q3).

^2^ Pearson’s Chi-squared test; Welch Two Sample t-test; Wilcoxon rank sum test.

ASMI, appendicular skeletal muscle mass index; WHtR, waist−to−height ratio; BMI, body mass index; SBP, systolic blood pressure; DBP, diastolic blood pressure; HR, heart rate; UACR, urinary albumin to creatinine ratio; LDL-C, low−density lipoprotein cholesterol; HDL-C, high-density lipoprotein cholesterol; ALT, alanine aminotransferase; Ca, calcium; HbA1c, glycated hemoglobin; DPN, diabetic peripheral neuropathy; DFU, diabetic foot ulcer; BMD, bone mineral density; DPP-4i, dipeptidyl peptidase-4 inhibitor; SGLT2i, sodium-glucose cotransporter 2 inhibitor; GLP-1RA, glucagon-like peptide-1 receptor agonist; DM, diabetes mellitus; eGFR, estimated Glomerular Filtration Rate.

### 3.2 Multivariable logistic regression analyses

Multivariable logistic regression was performed to identify factors associated with osteosarcopenia in patients with T2DM (**Table 2**). According to the VIF-based multicollinearity analysis (**Supplementary Table 2**), BMI and WHtR exhibited no evidence of collinearity (VIF < 5), which supported their simultaneous inclusion in the regression model. In Model 1, adjusted for BMI and z-WHtR, higher z-WHtR was associated with increased odds of osteosarcopenia (OR = 1.49, 95% CI: 1.32–1.68, p < 0.001), whereas higher BMI was associated with decreased odds (OR = 0.59, 95% CI: 0.57–0.62, p < 0.001). After further adjustment for gender, age, SBP, DBP, and HR in Model 2, these associations remained largely unchanged; male gender (OR = 2.55, 95% CI: 2.11–3.07, p < 0.001) and advanced age (OR = 1.06, 95% CI: 1.05–1.07, p < 0.001) emerged as significant independent risk factors. In the fully adjusted Model 3, male gender (OR = 2.34, 95% CI: 1.83–2.98, p < 0.001), age (OR = 1.06, 95% CI: 1.05–1.07, p < 0.001), fracture history (OR = 1.44, 95% CI: 1.05–1.97, p = 0.023), DFU (OR = 1.60, 95% CI: 1.08–2.38, p = 0.020), and DKD (OR = 1.23, 95% CI: 1.01–1.51, p = 0.043) were independently associated with elevated odds of osteosarcopenia, while higher BMI remained a protective factor (OR = 0.56, 95% CI: 0.53–0.59, p < 0.001). z-WHtR also retained a significant positive association (OR = 1.47, 95% CI: 1.28–1.69, p < 0.001). Smoking was not statistically significant (OR = 1.22, 95% CI: 0.96–1.54, p = 0.098).

**Table 2:** Multivariable logistic regression analysis for osteosarcopenia in T2DM patients.

Models	Characteristic	N	Event N	OR	95% CI	p-value
Model 1	BMI	5,412	890	0.59	0.57, 0.62	<0.001
	z-WHtR	5,412	890	1.49	1.32, 1.68	<0.001
Model 2	Gender					
	Female	2,552	321	—	—	
	Male	2,860	569	2.55	2.11, 3.07	<0.001
	Age	5,412	890	1.06	1.05, 1.07	<0.001
	BMI	5,412	890	0.56	0.54, 0.59	<0.001
	z-WHtR	5,412	890	1.48	1.29, 1.69	<0.001
Model 3	Gender					
	Female	2,552	321	—	—	
	Male	2,860	569	2.34	1.83, 2.98	<0.001
	Age	5,412	890	1.06	1.05, 1.07	<0.001
	BMI	5,412	890	0.56	0.53, 0.59	<0.001
	z-WHtR	5,412	890	1.47	1.28, 1.69	<0.001
	Fracture					
	No	5,005	807	—	—	
	Yes	407	83	1.44	1.05, 1.97	0.023
	DFU					
	No	5,201	833	—	—	
	Yes	211	57	1.60	1.08, 2.38	0.020
	DKD					
	No	3,644	581	—	—	
	Yes	1,768	309	1.23	1.01, 1.51	0.043
	Smoking					
	No	3,974	575	—	—	
	Yes	1,438	315	1.22	0.96, 1.54	0.098

Model 1 was additionally adjusted for BMI, z-WHtR.

Model 2 was additionally adjusted for gender, age, SBP, DBP, and HR based on Model 1.

Model 3 was additionally adjusted for TG, HDL−C, HDL−C, UA, ALT, Alb, ALP, Ca, Fasting blood glucose, HbA1c, hypertension, CHD, fracture, DR, DPN, DFU, DKD, metformin, sulfonylureas, non−sulfonylurea drugs, glucosidase inhibitors, thiazolidinediones, DPP-4i, SGLT2i, GLP-1RA, insulin, statins, smoking, and drinking on the basis of Model 2.

CI, confidence interval; OR, odds ratio; z-WHtR, waist-to-height ratio after Z-score standardization; BMI, body mass index; SBP, systolic blood pressure; DBP, diastolic blood pressure; HR, heart rate; triglycerides; LDL-C, low−density lipoprotein cholesterol; HDL-C, high−density lipoprotein cholesterol; UA, uric acid; Alb, albumin; ALP, alkaline phosphatase; Ca, calcium; HbA1c, glycated hemoglobin; CHD, coronary heart disease; DR, diabetic retinopathy; DPN, diabetic peripheral neuropathy; DFU, diabetic foot ulcer; DKD, diabetic kidney disease; DPP-4i, dipeptidyl peptidase-4 inhibitor; SGLT2i, sodium-glucose cotransporter 2 inhibitor; GLP-1RA, glucagon-like peptide-1 receptor agonist; T2DM, type 2 diabetes mellitus.

### 3.3 Nonlinear associations of BMI and WHtR with osteosarcopenia

**Figure 1:**
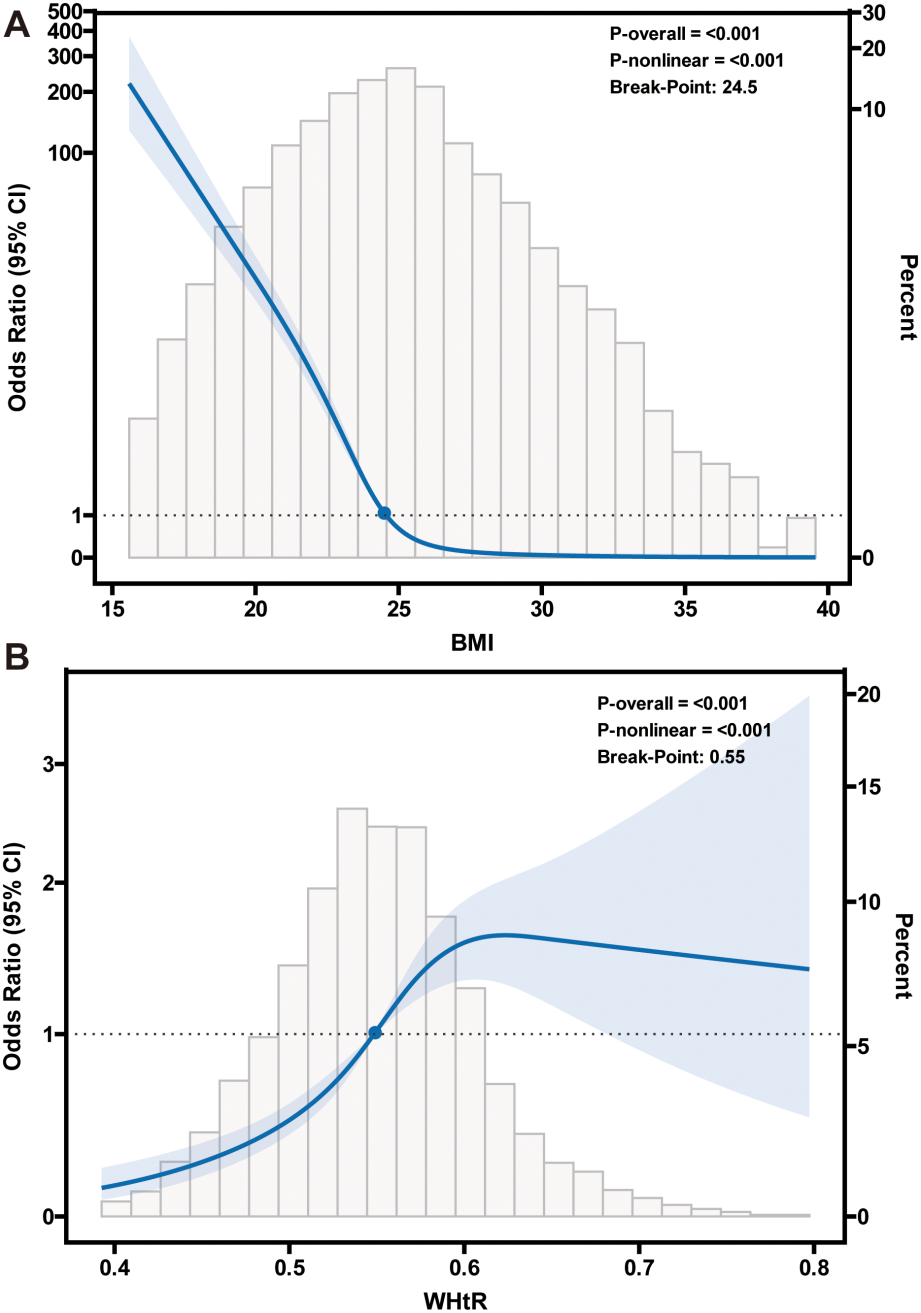
Nonlinear relationships of BMI and WHtR with osteosarcopenia assessed by restricted cubic spline (RCS) regression. RCS analysis of BMI **(A)** and WHtR **(B)** with osteosarcopenia adjusted for gender, age, SBP, DBP, HR, TG, LDL-C, HDL-C, ALT, UA, Alb, ALP, Ca, FBG, HbA1c, hypertension, CHD, fracture, DKD, DR, DPN, DFU, metformin, sulfonylureas, non-sulfonylurea drugs, glucosidase inhibitors, thiazolidinediones, DPP-4i, SGLT2i, GLP-1RA, insulin, statins, smoking, and drinking. The solid line represents the odds ratio, and the shaded area represents the 95% CI. CI, confidence interval; WHtR, waist-to-height ratio; BMI, body mass index; SBP, systolic blood pressure; DBP, diastolic blood pressure; HR, heart rate; triglycerides; TG, Triglyceride; LDL-C, low−density lipoprotein cholesterol; HDL-C, high−density lipoprotein cholesterol; UA, uric acid; Alb, albumin; ALP, alkaline phosphatase; Ca, calcium; FBG, fasting blood glucose; HbA1c, glycated hemoglobin; CHD, coronary heart disease; DR, diabetic retinopathy; DPN, diabetic peripheral neuropathy; DFU, diabetic foot ulcer; DKD, diabetic kidney disease; DPP-4i, dipeptidyl peptidase-4 inhibitor; SGLT2i, sodium-glucose cotransporter 2 inhibitor; GLP-1RA, glucagon-like peptide-1 receptor agonist.

RCS logistic regression was conducted to assess potential nonlinear relationships between BMI, WHtR, and osteosarcopenia, with adjustment for all covariates. As illustrated in **Figure 1**, a significant negative nonlinear relationship was detected between BMI and osteosarcopenia (P-overall < 0.001, P-nonlinear < 0.001, **Figure 1A**), with an approximate breakpoint identified at BMI = 24.5 kg/m². Below this threshold, the probability of osteosarcopenia declined significantly as BMI increased. Nevertheless, this association plateaued as BMI approached 30 kg/m². By contrast, WHtR demonstrated a significant positive nonlinear association (P-overall < 0.001, P for nonlinearity < 0.001, **Figure 1B**). When WHtR exceeded 0.55, the corresponding OR rose above 1. As indicated by this result, WHtR acted as a significant risk factor for osteosarcopenia.

### 3.4 Machine learning

**Figure 2:**
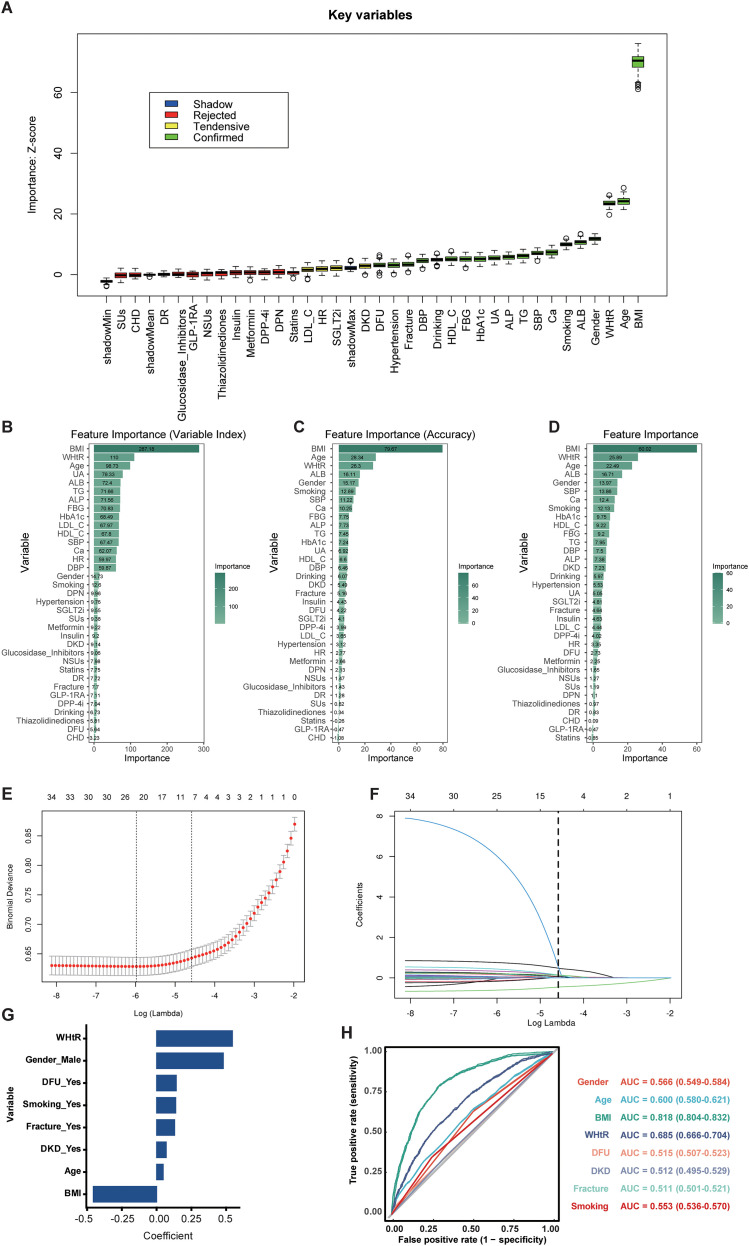
Machine learning-based identification and validation of key predictors for osteosarcopenia in in T2DM patients. **(A)** Boruta algorithm for feature selection, where green, yellow, and red boxes indicate confirmed, tentative, and rejected predictors, respectively, against shadow feature benchmarks (blue). **(B–D)** Random Forest variable importance rankings based on **(B)** Gini index, **(C)** accuracy, and **(D)** default model contribution. **(E–G)** LASSO regression analysis, including **(E)** tuning parameter (λ) selection via 10-fold cross-validation, **(F)** coefficient profile paths, and **(G)** histogram of the coefficients of the selected features. **(H)** ROC curve analysis comparing the predictive accuracy (AUC) and 95% CIs of individual variables. CI, confidence interval; WHtR, waist-to-height ratio; BMI, body mass index; SBP, systolic blood pressure; DBP, diastolic blood pressure; HR, heart rate; triglycerides; LDL-C, low−density lipoprotein cholesterol; HDL-C, high−density lipoprotein cholesterol; BUN, blood urea nitrogen; UA, uric acid; Alb, albumin; ALP, alkaline phosphatase; Ca, calcium; FBG, Fasting blood glucose; HbA1c, glycated hemoglobin; CHD, coronary heart disease; DR, diabetic retinopathy; DPN, diabetic peripheral neuropathy; DFU, diabetic foot ulcer; DKD, diabetic kidney disease; DPP-4i, dipeptidyl peptidase-4 inhibitor; SGLT2i, sodium-glucose cotransporter 2 inhibitor; GLP-1RA, glucagon-like peptide-1 receptor agonist; SUs, sulfonylureas; NSUs, non-sulfonylurea drugs.

Three complementary machine learning strategies were integrated to identify the most informative predictors, with the aim of developing a parsimonious predictive model for osteosarcopenia in T2DM patients. First, the Boruta algorithm was applied for initial feature selection, which identified BMI, age, WHtR, gender, Alb, smoking, and Ca as the most significant predictors (**Figure 2A**). Subsequently, random forest analysis was utilized to evaluate variable importance based on three distinct metrics: the Gini index, accuracy-based ranking, and the model’s default contribution measure. This analysis consistently identified WHtR, BMI, age, gender, and smoking as pivotal features across all evaluation frameworks, verifying their robustness (**Figures 2B–D**). LASSO regression was implemented for mitigating potential multicollinearity and optimizing the feature set. With the optimal penalty parameter (λ), LASSO performed feature shrinkage. A final set of key predictors was selected, including WHtR, BMI, gender, age, DKD, DFU, smoking, and fracture history (**Figures 2E–G**). ROC analysis was further conducted to assess the predictive performance of individual variables, including BMI (AUC = 0.818), WHtR (AUC = 0.685), age (AUC = 0.600), gender (AUC = 0.566), smoking (AUC = 0.553), DFU (AUC = 0.515), DKD (AUC = 0.512), as well as fracture (AUC = 0.511) (**Figure 2H**).

### 3.5 Nomogram development and validation

**Figure 3:**
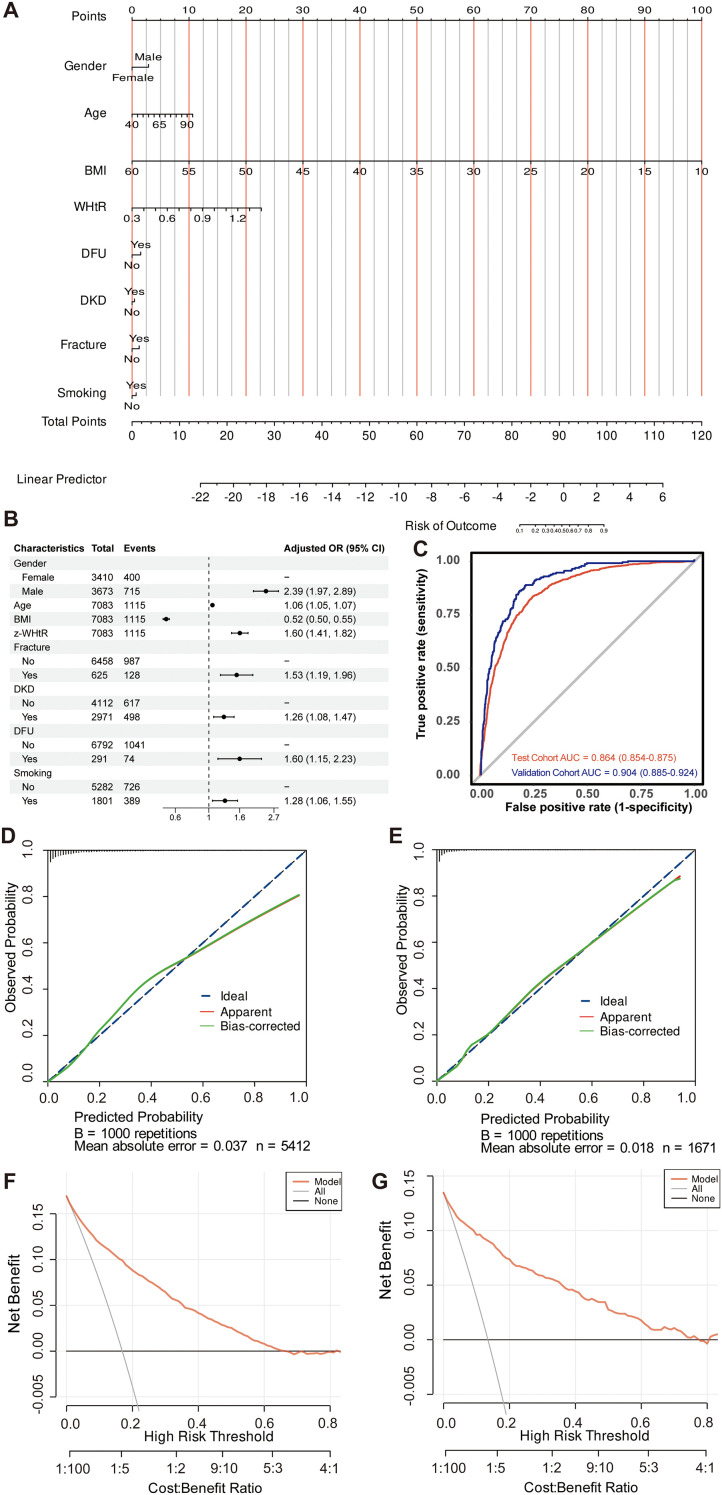
Development and validation of a nomogram for predicting osteosarcopenia risk in T2DM patients. **(A)** The nomogram integrating eight predictors. **(B)** Forest plot showing the odds ratios (ORs) and 95% confidence intervals (CIs) from the multivariable logistic regression analysis for each predictor. **(C)** Receiver operating characteristic (ROC) curves of the nomogram for the test cohort and validation cohort. **(D, E)** Calibration plots for the test cohort **(D)** and validation cohort **(E)**. **(F, G)** Decision curves (DCA) demonstrating the net clinical benefit of the nomogram in the test cohort **(F)** and validation cohort **(G)**. WHtR, waist-to-height ratio; BMI, body mass index; DFU, diabetic foot ulcer; DKD, diabetic kidney disease.

The final logistic regression model for the test cohort incorporated eight independent predictors, gender, age, BMI, WHtR, history of fracture, DFU, smoking status, and DKD, and was visualized as a nomogram (**Figure 3A**). Notably, BMI, WHtR, and age contributed the greatest predictive weight for osteosarcopenia. In this parsimonious model, all eight predictors achieved statistical significance (**Figure 3B**). Male gender was significantly associated with an elevated risk of osteosarcopenia relative to female gender (OR = 2.39, 95% CI: 1.97–2.89). Additional significant positive predictors included advanced age (OR = 1.06 per year, 95% CI: 1.05–1.07), DFU (OR = 1.60, 95% CI: 1.15–2.23), DKD (OR = 1.26, 95% CI: 1.08–1.47), prior fracture history (OR = 1.53, 95% CI: 1.19–1.96), and smoking status (OR = 1.28, 95% CI: 1.06–1.55). In contrast, higher BMI was associated with a reduced osteosarcopenia risk (OR = 0.52, 95% CI: 0.50–0.55), while z-WHtR showed a strong positive association with osteosarcopenia (OR = 1.60, 95% CI: 1.41–1.82). ROC analysis revealed that the predictive model exhibited excellent discriminative ability for osteosarcopenia in both cohorts. The AUC was 0.864 (95% CI: 0.854–0.875) for the test cohort and 0.904 (95% CI: 0.885–0.924) for the temporal validation cohort (**Figure 3C**). Calibration plots for both cohorts demonstrated good consistency between the observed and predicted probabilities of osteosarcopenia (**Figures 3D, E**). DCA of the two cohorts further confirmed that the nomogram yields favorable net clinical benefits, supporting its potential utility in clinical practice (**Figures 3F, G**).

## 4 Discussion

This study successfully developed and temporally validated a nomogram for predicting osteosarcopenia risk in patients with T2DM aged ≥ 40 years. By combining logistic regression and machine learning feature selection methods, we identified eight predictors, gender, age, BMI, WHtR, fracture history, DFU, smoking, and DKD, and integrated them into the predictive nomogram. The model achieved excellent discriminative performance, with an AUC of 0.864. Collectively, this nomogram serves as a practical and cost-effective tool for the early identification of T2DM patients at high risk of osteosarcopenia. It facilitates targeted population screening and timely clinical intervention, which may reduce the future burden of fractures and disability.

Our study demonstrated that a higher BMI serves as a robust protective factor against osteosarcopenia (OR = 0.56), while a higher WHtR acts as a significant independent risk factor (OR = 1.47). This divergent association remained significant even after rigorous multivariable adjustment, underscoring a typical “obesity paradox” in this population (16, 28). Nonlinear analyses further substantiated the heterogeneous effects of these two metrics: a distinct inflection point for BMI was identified at 24.5 kg/m^2^, below which the risk escalates rapidly; conversely, WHtR presents a threshold at 0.55, beyond which the risk rises sharply. This threshold is closely aligned with the central obesity cutoff (WHtR > 0.50) defined by the European Association for the Study of Obesity (EASO) (29), suggesting that stricter visceral adiposity management is paramount for preserving musculoskeletal health in patients with T2DM. The biological underpinnings of this paradox may lie in the fact that although a higher BMI reflects greater nutritional reserves (30), increased mechanical loading on bone (31), and a protective cushioning effect of adipose tissue against falls (15), it is limited by its inability to distinguish skeletal muscle mass from fat distribution (32). In contrast, visceral fat accumulation, as indicated by WHtR, not only serves as a core trigger for systemic metabolic disorders but also acts as a key pathophysiological hub that mediates and exacerbates the onset and progression of sarcopenia and osteoporosis in T2DM patients. As a highly active endocrine and inflammatory organ, visceral fat secretes abundant pro-inflammatory cytokines such as TNF-α and IL-6 while suppressing the expression of anti-inflammatory factors including adiponectin, thereby inducing systemic low-grade chronic inflammation and aggravating insulin resistance (33). Long-term chronic inflammation can directly activate skeletal muscle protein breakdown pathways and inhibit anabolic protein synthesis, ultimately leading to reduced muscle mass and strength (34). It can also enhance osteoclast activity and suppress osteoblast proliferation and differentiation, resulting in excessive bone resorption relative to bone formation and accelerated bone loss (35, 36). Furthermore, imbalanced secretion of adipokines including leptin and adiponectin can further disrupt the metabolic homeostasis of bone and skeletal muscle (37). Collectively, this “dual effect” indicates that relying solely on BMI to evaluate musculoskeletal health in T2DM patients is highly misleading. It is therefore imperative to integrate WHtR-based adiposity quality assessment into routine risk stratification to enable more precise clinical interventions.

This study found that male T2DM patients exhibited a significantly higher risk of osteosarcopenia. Consistently, Yu et al. developed gender-specific nomograms to predict sarcopenia in T2DM patients with osteoporosis and confirmed that the prevalence of sarcopenia is significantly higher in male patients than in female patients based on a cohort of 847 participants (38). The mechanistic basis underlying this male predominance is multifactorial. Androgen deficiency, which affects one-third to two-thirds of men with T2DM, arises from chronic hyperglycemia- and insulin resistance-induced suppression of the hypothalamic-pituitary-gonadal axis and acts as a key driver that exacerbates both metabolic dysregulation and musculoskeletal decline (39). Reduced androgen levels are correlated with decreased BMD and an increased fracture risk in males (40). Furthermore, gender-specific disparities in body composition may also account for this phenotypic difference. Under T2DM pathological conditions, males with higher baseline muscle mass endure a greater metabolic burden, given that muscle mass maintenance relies on intact insulin signaling; however, insulin resistance exerts more severe inhibitory effects on muscle protein synthesis in males (41). Smoking was identified as a modifiable risk factor for osteosarcopenia in our study. Although its association did not reach statistical significance in the fully adjusted Model 3, this is likely attributable to over-adjustment by numerous covariates that diluted the effect estimate. The consistency across machine learning approaches and the final parsimonious model supports smoking as a clinically meaningful predictor, warranting its inclusion in the nomogram. This result is corroborated by existing evidence that tobacco use increases oxidative stress and insulin resistance, two pathological conditions that impair muscle protein synthesis and compromise bone health (42, 43). Notably, the prevalence of smoking is considerably higher in males than in females, which may partially explain the elevated osteosarcopenia risk observed in male T2DM patients (44). A meta-analysis encompassing 2,904 participants revealed that diabetic nephropathy increases the risk of sarcopenia by 2.54-fold among T2DM patients (OR = 2.54, P = 0.001) (45). A nationwide Asian cohort study focusing on patients with newly diagnosed T2DM further demonstrated that DKD is associated with a mild increase in osteoporosis risk (adjusted hazard ratio: 1.14, 95% CI: 1.09–1.19) (46). These findings are highly consistent with our observation that DKD independently contributes to the pathogenesis of osteosarcopenia in T2DM patients. The underlying pathophysiological mechanisms linking DKD to osteosarcopenia involve complex interactions among disturbed mineral metabolism (including dysregulation of calcium, phosphate, parathyroid hormone, and vitamin D), chronic inflammation, anabolic resistance, and endocrine dysfunction, which collectively accelerate bone loss and muscle deterioration (47). The present study also identified prominent independent associations of DFU (OR = 1.60) and fracture history (OR = 1.44) with osteosarcopenia. Such associations can be explained by disuse atrophy. These diabetic complications severely limit physical activity, resulting in rapid physical deconditioning and muscle wasting, which is further aggravated by the catabolic state induced by diabetes (48). T2DM patients present an elevated fracture risk despite normal or increased BMD, a phenomenon attributable to impaired bone quality. This pathological alteration is driven by AGE-induced collagen cross-linking and can be reflected by a reduced trabecular bone score, independent of BMD (11, 49, 50). These findings collectively suggest that prior fracture events may serve as clinical indicators of underlying bone quality deterioration, warranting increased vigilance for osteosarcopenia in T2DM patients with a history of fragility fractures. DFU may act as a clinical proxy for a more severe and long-standing state of metabolic dysregulation and inflammatory burden that accelerates the development of osteosarcopenia (51). This underscores the need for comprehensive musculoskeletal screening in all T2DM patients presenting with diabetic foot complications. Furthermore, the nomogram exhibited strong predictive performance, with an AUC of 0.864 in the test cohort and 0.904 in the validation cohort, positioning it as a potentially valuable clinical tool. By relying solely on routinely collected clinical parameters (gender, age, BMI, WHtR, fracture history, DFU, smoking, and DKD), it enables rapid risk stratification without the need for specialized assessments such as DXA. This model could be particularly useful in primary care or endocrinology clinics to identify T2DM patients who would benefit most from confirmatory DXA and functional assessment for osteosarcopenia.

Several limitations should be acknowledged. First, the retrospective design and the use of a hospitalized cohort may introduce selection bias, limiting the generalizability of the findings to the broader T2DM population. Second, sarcopenia was defined using low ASMI combined with low grip strength rather than the full AWGS 2019 criteria due to substantial missing gait speed data; although the updated AWGS 2025 consensus supports this simplified approach, the prevalence of osteosarcopenia may have been overestimated. Third, sarcopenia was defined using ALST/height², which applies fixed cut-offs that systematically underdiagnose low muscle mass in individuals with higher BMI (52), suggesting that the inverse BMI–osteosarcopenia association may partially reflect mathematical coupling rather than a purely biological effect. Although we incorporated WHtR to partially mitigate this bias, it does not fully eliminate the underlying coupling, and thus the apparent protective effect of BMI should be interpreted with caution. Fourth, key confounders such as glycemic variability, insulin dosage, and hypoglycemia history could not be assessed due to missing data. Fifth, although antidiabetic medications (including thiazolidinediones, GLP-1RA, and SGLT2i) were incorporated into the regression model, residual confounding by medication use cannot be completely excluded. Finally, although temporal validation was performed using a cohort from the same institution, external validation in an independent multicenter cohort is lacking. Future prospective, multicenter studies are warranted to validate our findings.

## 5 Conclusion

This study developed and validated a nomogram for predicting osteosarcopenia risk in T2DM patients aged ≥ 40 years using eight readily available variables (gender, age, BMI, WHtR, fracture history, DFU, smoking, and DKD). The model demonstrates excellent discrimination and good calibration, offering a practical tool for early risk stratification. Key findings highlight the protective role of higher BMI and the detrimental effect of central adiposity (WHtR). Further multicenter external validation based on prospective multicenter cohorts is warranted.

## Data Availability

The raw data supporting the conclusions of this article will be made available by the authors, without undue reservation.

## Supplementary material

Supplementary Table 1 Baseline characteristics of T2DM patients in the validation cohort. ^1^n (%); Median (Q1, Q3). ^2^Pearson’s Chi-squared test; Wilcoxon rank sum test. ASMI, appendicular skeletal muscle mass index; WHtR, waist−to−height ratio; BMI, body mass index; SBP, systolic blood pressure; DBP, diastolic blood pressure; HR, heart rate; UACR, urinary albumin to creatinine ratio; LDL-C, low−density lipoprotein cholesterol; HDL-C, high-density lipoprotein cholesterol; ALT, alanine aminotransferase; Ca, calcium; HbA1c, glycated hemoglobin; DPN, diabetic peripheral neuropathy; DFU, diabetic foot ulcer; BMD, bone mineral density; DPP-4i, dipeptidyl peptidase-4 inhibitor; SGLT2i, sodium-glucose cotransporter 2 inhibitor; GLP-1RA, glucagon-like peptide-1 receptor agonist; DM, diabetes mellitus; eGFR, estimated Glomerular Filtration Rate.

Supplementary Table 2 Variance inflation factor (VIF) for covariates. WHtR, waist−to−height ratio; BMI, body mass index; SBP, systolic blood pressure; DBP, diastolic blood pressure; HR, heart rate; LDL-C, low−density lipoprotein cholesterol; ALT, alanine aminotransferase; Ca, calcium; HbA1c, glycated hemoglobin; DPN, diabetic peripheral neuropathy; DFU, diabetic foot ulcer; DPP-4i, dipeptidyl peptidase-4 inhibitor; SGLT2i, sodium-glucose cotransporter 2 inhibitor; GLP-1RA, glucagon-like peptide-1 receptor agonist; SE, standard error; CI, confidence interval.
