# Supplementary material for: A nomogram integrating machine learning with clinical predictors for osteosarcopenia risk prediction in type 2 diabetes mellitus
Source: Front Endocrinol (Lausanne). 2026 Jul 15;17:1876521. doi: 10.3389/fendo.2026.1876521 (PMC13414204; doi:10.3389/fendo.2026.1876521)
Supplement: Supplementary Table 1 — Baseline characteristics of T2DM patients in the validation cohort. 1n (%); Median (Q1, Q3). 2Pearson’s Chi-squared test; Wilcoxon rank sum test. ASMI, appendicular skeletal muscle mass index; WHtR, waist−to−height ratio; BMI, body mass index; SBP, systolic blood pressure; DBP, diastolic blood pressure; HR, heart rate; UACR, urinary albumin to creatinine ratio; LDL-C, low−density lipoprotein cholesterol; HDL-C, high-density lipoprotein cholesterol; ALT, alanine aminotransferase; Ca, calcium; HbA1c, glycated hemoglobin; DPN, diabetic peripheral neuropathy; DFU, diabetic foot ulcer; BMD, bone mineral density; DPP-4i, dipeptidyl peptidase-4 inhibitor; SGLT2i, sodium-glucose cotransporter 2 inhibitor; GLP-1RA, glucagon-like peptide-1 receptor agonist; DM, diabetes mellitus; eGFR, estimated Glomerular Filtration Rate. [file Table1.docx]

Table S1 Baseline characteristics of T2DM patients in the validation cohort

| **Characteristic** | **Osteosarcopenia** | | | **P-value^2^** |
| --- | --- | --- | --- | --- |
|  | **Overall  N = 1,671^1^** | **No  N = 1,446^1^** | **Yes  N = 225^1^** |  |
| **Gender** |  |  |  | <0.001 |
| Female | 858 (51.3%) | 779 (53.9%) | 79 (35.1%) |  |
| Male | 813 (48.7%) | 667 (46.1%) | 146 (64.9%) |  |
| **Age (year)** | 66 (59, 73) | 65 (58, 73) | 69 (63, 76) | <0.001 |
| **ASMI (kg/m^2^)** | 6.46 (5.87, 7.25) | 6.59 (5.99, 7.39) | 5.45 (4.90, 6.27) | <0.001 |
| **Grip strength (kg)** | 27 (21, 32) | 28 (23, 32) | 20 (16, 24) | <0.001 |
| **Waist circumference (cm)** | 88 (82, 94) | 89 (83, 95) | 80 (74, 86) | <0.001 |
| **Height (cm)** | 162 (156, 169) | 162 (156, 169) | 162 (156, 168) | 0.613 |
| **BMI (kg/m^2^)** | 25.6 (23.1, 28.0) | 26.2 (24.1, 28.5) | 21.3 (19.4, 23.0) | <0.001 |
| **WHtR** | 0.54 (0.51, 0.59) | 0.55 (0.51, 0.59) | 0.50 (0.46, 0.54) | <0.001 |
| **SBP (mmHg)** | 130 (119, 144) | 129 (118, 144) | 134 (123, 142) | 0.137 |
| **DBP (mmHg)** | 73 (65, 81) | 73 (65, 81) | 74 (64, 82) | 0.241 |
| **HR (bpm)** | 78 (69, 85) | 78 (69, 85) | 75 (67, 86) | 0.661 |
| **UACR (mg/g)** | 79 (35, 254) | 77 (34, 254) | 90 (40, 261) | 0.139 |
| **Triglycerides (mmol/L)** | 1.31 (0.92, 1.77) | 1.33 (0.95, 1.78) | 1.20 (0.77, 1.57) | <0.001 |
| **Total cholesterol (mmol/L)** | 4.05 (3.36, 4.74) | 4.06 (3.38, 4.74) | 4.03 (3.35, 4.70) | 0.650 |
| **LDL cholesterol (mmol/L)** | 2.49 (1.83, 3.12) | 2.48 (1.83, 3.12) | 2.53 (1.87, 3.07) | 0.819 |
| **HDL cholesterol (mmol/L)** | 1.11 (0.94, 1.30) | 1.11 (0.94, 1.29) | 1.12 (0.92, 1.47) | 0.262 |
| **Blood urea nitrogen (U/L)** | 6.10 (5.26, 7.80) | 6.10 (5.34, 7.80) | 6.05 (4.84, 8.01) | 0.323 |
| **Uric acid (μmol/L)** | 310 (258, 363) | 309 (258, 363) | 317 (258, 363) | 0.941 |
| **Albumin(g/L)** | 41.5 (39.1, 43.1) | 41.5 (39.2, 43.1) | 41.4 (39.1, 43.0) | 0.565 |
| **Alkaline phosphatase (U/L)** | 68 (51, 85) | 68 (50, 85) | 70 (55, 91) | 0.011 |
| **Ca (mmol/L)** | 2.23 (2.16, 2.28) | 2.23 (2.16, 2.28) | 2.22 (2.15, 2.29) | 0.244 |
| **Fasting glucose (mmol/L)** | 6.14 (5.01, 7.84) | 6.14 (5.01, 7.72) | 6.26 (5.04, 8.34) | 0.582 |
| **HbA1c (%)** | 5.80 (4.70, 7.50) | 5.80 (4.70, 7.40) | 6.00 (4.70, 8.00) | 0.572 |
| **Creatinine (μmol/L)** | 69 (56, 83) | 69 (56, 83) | 72 (56, 87) | 0.174 |
| **eGFR (mL/min/1.73m²)** | 91 (74, 100) | 90 (75, 100) | 91 (72, 100) | 0.930 |
| **Hypertension, n (%)** | 1,080 (64.6%) | 941 (65.1%) | 139 (61.8%) | 0.336 |
| **Coronary heart disease, n (%)** | 375 (22.4%) | 308 (21.3%) | 67 (29.8%) | 0.005 |
| **Fracture, n (%)** | 218 (13.0%) | 173 (12.0%) | 45 (20.0%) | <0.001 |
| **Duration of DM (years)** | 11 (6, 20) | 11 (6, 20) | 11 (6, 19) | 0.588 |
| **Diabetic kidney disease, n (%)** | 1,203 (72.0%) | 1,014 (70.1%) | 189 (84.0%) | <0.001 |
| **Diabetic retinopathy, n (%)** | 640 (38.3%) | 570 (39.4%) | 70 (31.1%) | 0.017 |
| **DPN, n (%)** | 720 (43.1%) | 630 (43.6%) | 90 (40.0%) | 0.315 |
| **DFU, n (%)** | 80 (4.8%) | 63 (4.4%) | 17 (7.6%) | 0.037 |
| **Metformin, n (%)** | 1,491 (89.2%) | 1,283 (88.7%) | 208 (92.4%) | 0.094 |
| **Sulfonylureas, n (%)** | 114 (6.8%) | 95 (6.6%) | 19 (8.4%) | 0.300 |
| **Non-sulfonylurea drugs, n (%)** | 162 (9.7%) | 140 (9.7%) | 22 (9.8%) | 0.964 |
| **Glucosidase Inhibitors, n (%)** | 702 (42.0%) | 599 (41.4%) | 103 (45.8%) | 0.218 |
| **Thiazolidinediones, n (%)** | 45 (2.7%) | 40 (2.8%) | 5 (2.2%) | 0.639 |
| **Insulin, n (%)** | 687 (41.1%) | 586 (40.5%) | 101 (44.9%) | 0.216 |
| **DPP-4i, n (%)** | 321 (19.2%) | 276 (19.1%) | 45 (20.0%) | 0.746 |
| **SGLT2i, n (%)** | 555 (33.2%) | 461 (31.9%) | 94 (41.8%) | 0.003 |
| **GLP-1RA, n (%)** | 107 (6.4%) | 99 (6.8%) | 8 (3.6%) | 0.061 |
| **Statins, n (%)** | 1,233 (73.8%) | 1,074 (74.3%) | 159 (70.7%) | 0.252 |
| **Statins, n (%)** | 1,233 (73.8%) | 1,074 (74.3%) | 159 (70.7%) | 0.252 |
| **Smoking, n (%)** | 363 (21.7%) | 289 (20.0%) | 74 (32.9%) | <0.001 |
| **Drinking, n (%)** | 171 (10.2%) | 134 (9.3%) | 37 (16.4%) | <0.001 |
| **BMD category, n (%)** |  |  |  | <0.001 |
| Normal | 1,148 (68.7%) | 1,148 (79.4%) | 0 (0.0%) |  |
| Osteopenia | 330 (19.7%) | 194 (13.4%) | 136 (60.4%) |  |
| Osteoporosis | 193 (11.5%) | 104 (7.2%) | 89 (39.6%) |  |
| **Sarcopenia, n (%)** | 488 (29.2%) | 263 (18.2%) | 225 (100.0%) | <0.001 |
| ^1^n (%); Median (Q1, Q3) | | | | |
| ^2^Pearson's Chi-squared test; Wilcoxon rank sum test  Abbreviations: ASMI, appendicular skeletal muscle mass index; WHtR, waist‑to‑height ratio; BMI, body mass index; SBP, systolic blood pressure; DBP, diastolic blood pressure; HR, heart rate; UACR, urinary albumin to creatinine ratio; LDL-C, low‑density lipoprotein cholesterol; HDL-C, high-density lipoprotein cholesterol; ALT, alanine aminotransferase; Ca, calcium; HbA1c, glycated hemoglobin; DPN, diabetic peripheral neuropathy; DFU, diabetic foot ulcer; BMD, bone mineral density; DPP-4i, dipeptidyl peptidase-4 inhibitor; SGLT2i, sodium-glucose cotransporter 2 inhibitor; GLP-1RA, glucagon-like peptide-1 receptor agonist; DM, diabetes mellitus; eGFR, estimated Glomerular Filtration Rate. | | | | |
