# Supplementary material for: A nomogram integrating machine learning with clinical predictors for osteosarcopenia risk prediction in type 2 diabetes mellitus
Source: Front Endocrinol (Lausanne). 2026 Jul 15;17:1876521. doi: 10.3389/fendo.2026.1876521 (PMC13414204; doi:10.3389/fendo.2026.1876521)
Supplement: Supplementary Table 2 — Variance inflation factor (VIF) for covariates. WHtR, waist−to−height ratio; BMI, body mass index; SBP, systolic blood pressure; DBP, diastolic blood pressure; HR, heart rate; LDL-C, low−density lipoprotein cholesterol; ALT, alanine aminotransferase; Ca, calcium; HbA1c, glycated hemoglobin; DPN, diabetic peripheral neuropathy; DFU, diabetic foot ulcer; DPP-4i, dipeptidyl peptidase-4 inhibitor; SGLT2i, sodium-glucose cotransporter 2 inhibitor; GLP-1RA, glucagon-like peptide-1 receptor agonist; SE, standard error; CI, confidence interval. [file Table2.docx]

Table S2. Variance inflation factor (VIF) for covariates.

| Term | VIF | CI_95_VIF | SE_factor |
| --- | --- | --- | --- |
| Gender | 1.945 | 1.871, 2.024 | 1.395 |
| Age | 1.534 | 1.482, 1.592 | 1.239 |
| BMI | 1.998 | 1.922, 2.081 | 1.414 |
| WHtR | 2.053 | 1.974, 2.138 | 1.433 |
| SBP | 1.879 | 1.809, 1.955 | 1.371 |
| DBP | 1.697 | 1.637, 1.764 | 1.303 |
| HR | 1.111 | 1.083, 1.149 | 1.054 |
| LDL-C | 4.658 | 4.444, 4.884 | 2.158 |
| HDL-C | 1.7 | 1.64, 1.767 | 1.304 |
| Uric acid | 1.345 | 1.303, 1.393 | 1.16 |
| Triglycerides | 1.952 | 1.878, 2.032 | 1.397 |
| Total cholesterol | 6.257 | 5.961, 6.57 | 2.501 |
| Albumin | 2.115 | 2.033, 2.204 | 1.454 |
| Alkaline phosphatase | 1.121 | 1.092, 1.159 | 1.059 |
| Ca | 1.785 | 1.72, 1.856 | 1.336 |
| Fasting blood glucose | 1.428 | 1.381, 1.48 | 1.195 |
| HbA1c | 1.584 | 1.53, 1.645 | 1.259 |
| ALT | 1.116 | 1.088, 1.154 | 1.056 |
| Hypertension | 1.378 | 1.334, 1.427 | 1.174 |
| Coronary heart disease | 1.068 | 1.044, 1.106 | 1.034 |
| Fracture | 1.048 | 1.027, 1.088 | 1.024 |
| Diabetic retinopathy | 1.175 | 1.143, 1.215 | 1.084 |
| DPN | 1.13 | 1.1, 1.168 | 1.063 |
| DFU | 1.061 | 1.038, 1.099 | 1.03 |
| Diabetic kidney disease | 1.268 | 1.231, 1.312 | 1.126 |
| Metformin | 1.164 | 1.133, 1.204 | 1.079 |
| Sulfonylureas | 1.119 | 1.091, 1.157 | 1.058 |
| Non sulfonylurea drugs | 1.084 | 1.058, 1.122 | 1.041 |
| Glucosidase inhibitors | 1.155 | 1.124, 1.194 | 1.075 |
| Thiazolidinediones | 1.08 | 1.054, 1.117 | 1.039 |
| Insulin | 1.124 | 1.095, 1.162 | 1.06 |
| Statins | 1.344 | 1.302, 1.392 | 1.159 |
| Smoking | 1.625 | 1.569, 1.688 | 1.275 |
| Drinking | 1.32 | 1.279, 1.366 | 1.149 |
| DPP-4i | 1.386 | 1.342, 1.436 | 1.177 |
| SGLT2 | 1.358 | 1.315, 1.406 | 1.165 |
| GLP-1RA | 1.039 | 1.019, 1.081 | 1.019 |

Abbreviations: WHtR, waist‑to‑height ratio; BMI, body mass index; SBP, systolic blood pressure; DBP, diastolic blood pressure; HR, heart rate; LDL-C, low‑density lipoprotein cholesterol; ALT, alanine aminotransferase; Ca, calcium; HbA1c, glycated hemoglobin; DPN, diabetic peripheral neuropathy; DFU, diabetic foot ulcer; DPP-4i, dipeptidyl peptidase-4 inhibitor; SGLT2i, sodium-glucose cotransporter 2 inhibitor; GLP-1RA, glucagon-like peptide-1 receptor agonist; SE, standard error; CI, confidence interval.
